# RIP140 regulates *POLK* gene expression and the response to alkylating drugs in colon cancer cells 

**DOI:** 10.20517/cdr.2021.133

**Published:** 2022-05-07

**Authors:** Pascale Palassin, Marion Lapierre, Sandrine Bonnet, Marie-Jeanne Pillaire, Balázs Győrffy, Catherine Teyssier, Stéphan Jalaguier, Jean-Sébastien Hoffmann, Vincent Cavaillès, Audrey Castet-Nicolas

**Affiliations:** ^1^IRCM, Institut de Recherche en Cancérologie de Montpellier, INSERM, U1194, Institut Régional du Cancer de Montpellier, Université de Montpellier, Montpellier 34298, France.; ^2^Institut de Pharmacologie et de Biologie Structurale, IPBS, Université de Toulouse, CNRS, UPS, Toulouse 31077, France.; ^3^Department of Bioinformatics, Semmelweis University and TTK Lendület Cancer Biomarker Research Group, Budapest H-1117, Hungary.; ^4^Laboratoire de pathologie, Laboratoire d’excellence Toulouse Cancer, Institut Universitaire du Cancer-Toulouse, Oncopole, Toulouse 31059, France.; ^5^Département de Pharmacie Clinique, Centre Hospitalo-Universitaire Montpellier, Montpellier 34295, France.; ^6^Unité de Formation et de Recherche des Sciences Pharmaceutiques et Biologiques, Montpellier 34090, France.; ^7^Should be considered as co-senior authors.

**Keywords:** Colorectal cancer, genome stability, translesion DNA synthesis polymerase, Pol Kappa, RIP140

## Abstract

**Aim:** The transcription factor RIP140 (receptor interacting protein of 140 kDa) is involved in intestinal tumorigenesis. It plays a role in the control of microsatellite instability (MSI), through the regulation of *MSH2* and *MSH6* gene expression. The aim of this study was to explore its effect on the expression of *POLK*, the gene encoding the specialized translesion synthesis (TLS) DNA polymerase κ known to perform accurate DNA synthesis at microsatellites.

**Methods:** Different mouse models and engineered human colorectal cancer (CRC) cell lines were used to analyze by RT-qPCR, while Western blotting and luciferase assays were used to elucidate the role of RIP140 on *POLK* gene expression. Published DNA microarray datasets were reanalyzed. The *in vitro* sensitivity of CRC cells to methyl methane sulfonate and cisplatin was determined.

**Results:** RIP140 positively regulates, at the transcriptional level, the expression of the *POLK* gene, and this effect involves, at least partly, the p53 tumor suppressor. In different cohorts of CRC biopsies (with or without MSI), a strong positive correlation was observed between *RIP140* and *POLK* gene expression. In connection with its effect on POLK levels and the TLS function of this polymerase, the cellular response to methyl methane sulfonate was increased in cells lacking the *Rip140* gene. Finally, the association of RIP140 expression with better overall survival of CRC patients was observed only when the corresponding tumors exhibited low levels of *POLK*, thus strengthening the functional link between the two genes in human CRC.

**Conclusion:** The regulation of *POLK* gene expression by RIP140 could thus contribute to the maintenance of microsatellite stability, and more generally to the control of genome integrity.

## INTRODUCTION

Colorectal cancer (CRC) is one of the most frequent cancers worldwide and genetic instability exerts a driving role in this malignancy^[1]^. The mismatch repair (MMR) system is one of the various cellular systems involved in the maintenance of genome integrity through the correction of mistakes that occur during DNA replication. Once impaired (as occurs in 2%-3% of CRC cases with an inherited component including Lynch syndrome^[2,3]^), it generates microsatellite instability (MSI) and a hypermutated tumor phenotype with a high frequency of point and frameshift mutations^[4,5]^.

In addition to molecular machineries that cope with DNA repair, mammalian cells possess enzymes with translesion DNA synthesis (TLS) activity. The *POLK* gene encodes Polκ, one of the Y-family TLS polymerases, which possesses unique DNA damage bypass capability^[6]^. Interestingly, despite any associated proofreading exonuclease activity, Polκ was shown to display high accuracy during dinucleotide microsatellite DNA synthesis^[7]^, and *Polκ*-/- mice display a spontaneous mutator phenotype in various tissues^[8]^. This could be linked to its less open active site compared to other inaccurate specialized DNA polymerases from the Y family^[9]^. In fact, Polκ possesses a unique structural feature, an extension of its N-terminal region, called N-clasp, that protrudes from the thumb domain and encircles DNA in proximity to a primer terminus^[10]^. This results in a much more stable complex once Polκ binds to a mismatched DNA substrate, facilitating the extension step across various minor groove distortion or lesion. Besides its role in MSI maintenance together with MMR^[7]^, Polκ seems to prevent DNA damage-induced toxic effects of methylnitrosourea, another process dependent on the MMR system^[11]^. This link between MMR and Polκ is reinforced by the findings that POLK interacts with MSH2^[12]^ and partially protects human cells from the MMR-dependent cytotoxicity of O6-methylguanine lesions^[11]^.

Our laboratory recently reported that the RIP140 (receptor interacting protein of 140 kDa) gene was involved in the normal and tumoral development of the intestinal epithelium. RIP140, also known as NRIP1 (nuclear receptor-interacting protein 1), was first identified as a transcriptional repressor of nuclear hormone receptors^[13,14]^. We and others then characterized RIP140 as a coregulator of various transcription factors, including, for instance, E2F^[15]^ or NFKB^[16]^. The repressive activity of RIP140 involves several inhibitory domains interacting with histone deacetylases^[17]^ and is controlled by different post-translational modifications^[18]^. Using a mouse model lacking the *Rip140* gene, various physiological processes were shown to be regulated by RIP140, including female fertility^[19] ^and mammary gland morphogenesis^[20]^, fat metabolism^[21]^, pro-inflammatory cytokine response^[22]^, or cognition^[23]^.

In the intestinal epithelium, our laboratory demonstrated that RIP140 inhibits the Wnt/β-catenin signaling pathway and, as a consequence, exerts an anti-proliferative effect^[24]^. In line with this result, we found that this transcription coregulator inhibits the Paneth cells lineage through the regulation of SOX9 expression and activity^[25]^. In addition, RIP140 expression decreased in CRC samples compared to the adjacent healthy tissue. In sporadic CRC, RIP140 mRNA and protein levels significantly correlated with better overall survival of patients and were identified as good prognosis markers^[24]^. More recently, we demonstrated that RIP140 was acting as a transcriptional regulator of *MSH2* and *MSH6* gene expression and was involved in the regulation of the MSI and hypermutator phenotype in CRC cells^[26]^. Interestingly, a frameshift mutation in the RIP140 coding sequence identified in MSI CRC tumors exhibits a dominant negative activity and correlates with shorter overall survival of patients with advanced CRC.

In the present study, we explored whether RIP140 could regulate POLK as a factor involved in microsatellite stability. We demonstrated that, in both engineered mouse models and human CRC cells, RIP140 positively regulates the *POLK* gene expression at the transcriptional level, at least partly *via* a p53-dependent mechanism. A strong correlation was observed between the expression of the *RIP140* and *POLK* genes in CRC biopsies. Moreover, cells knocked out for the *Rip140* gene were shown to be more sensitive to methyl methane sulfonate (MMS), a drug known to induce DNA lesions activating a POLK response. Finally, our data demonstrate a significant impact of *POLK* gene expression on the prognosis value of RIP140 in human CRC. We propose that the modulation of *POLK* gene expression by RIP140 could reinforce its effect on the maintenance of genome integrity and, more particularly, on the stability of microsatellite sequences.

## METHODS

### Plasmids

pRL-CMV-renilla and pGL promoters were obtained from Promega (Charbonnieres, France). pEF-cmyc-RIP140 was previously described^[27]^. pEGFP-RIP140 was a kind gift of Dr. Johanna Zilliacus, (Karolinska Institutet, Huddinge, Sweden)^[28]^. pEF-cmyc-RIP^MSI^ was generated by mutagenesis using the QuikChange® Site-Directed Mutagenesis Kit (Stratagene). pEF-cmyc-RIP^MSI^ was digested with AflII and EcoRV enzymes, and the resulting insert was cloned into pEGFP-RIP140 to create pEGFP-RIP^MSI^. GFP, GFP-RIP140, and GFP-RIP^MSI^ were PCR amplified and cloned into pTRIPZ previously digested with AgeI and MluI to create pTRIPZ-GFP, pTRIPZ-RIP140 and pTRIPZ-RIP^MSI^, respectively. All the engineered PCR constructs were sequenced.

### Cell culture and transfections

Mouse embryonic fibroblasts (MEFs) derived from wild-type or RIPKO mice previously described^[24]^, and the stably transfected MEFs described by Palassin *et al.*^[26]^ were grown in DMEM-F12 medium supplemented with 10% FCS, 100 U/mL penicillin, 100 mg/mL streptomycin, and 100 mg/mL sodium pyruvate, with 40 µg/mL puromycine for selection of stably transfected cells. HCT116, RKO, SW480, and HT29 human colon cancer cells were grown as previously described^[26]^ and stably transfected with the empty pEGFP vector (Clontech®) or with the same vector containing the full-length human RIP140 cDNA. The SW620 human cell line was grown identically. HCT116-GFP and HCT116-RIP140 cells were previously described^[24]^ and grown in McCoy medium and 750 μg/mL G418. Small interfering RNA (siRNA) transfections were performed using INTERFERin® on cells seeded the day before in a 6-well plate (3 × 10^5^ cells per well). Each transfection was performed in triplicate, and interference efficiencies were tested by quantitative RT-PCR.

### Animals

C57BL/6J Rip140^-/- ^(RIPKO) mice^[19]^ were given by Pr MG Parker (Imperial College London, London, UK). C57BL/6/129 RIP140 transgenic (RIPTg) mice were generated using the Speedy Mouse® Technology (Nucleis) by insertion of a single copy of the human RIP140 cDNA at the HPRT locus^[24]^. All animals were maintained under standard conditions, on a 12 h:12 h light/dark schedule and fed a chow diet ad libitum, according to European Union guidelines for the use of laboratory animals. In vivo experiments were performed in compliance with the French guidelines for experimental animal studies (agreement B34-172-27).

### Luciferase assays

HCT116 cells were plated in 96-well plates (2.5 × 10^4^ cells per well) 24 h prior to DNA transfection with Jet-PEI® (275 ng of total DNA). The pGL3-POLK Luc and its truncated mutant pGL3-83 vectors were previously described^[29]^. The pGL3-29 vector was constructed for this work and co-transfected in HCT116 cells. The pRL-CMV-renilla plasmid (Ozyme®) was used to normalize transfection efficiency. Firefly luciferase values were measured and normalized by the Renilla luciferase activity. Values were expressed as the mean ratio of luciferase activities.

### Cell proliferation and cytotoxicity assays

Cells were seeded in quadruplicate at a density of 2 × 10^3^ cells per well. At the indicated time, 0.5 mg/mL of 3-(4,5-dimethylthiazol-2-yl)-2,5-diphenyltetrazolium bromide (MTT) (Sigma-Aldrich®, St Louis, MO, USA) was added and incubated at 37 °C for 4 h. Formazan crystals were solubilized in DMSO and absorbance read at 560 nm on a spectrophotometer. The results were normalized to the cell density at Day 1. For cytotoxicity assays, cells were seeded in quadruplicate in a 96-well plate (2.5 × 10^3^ cells per well) and exposed the day after to increasing concentrations of cytotoxic drugs orto vehicle alone. The cells were exposed to the drug during six days and cell proliferation was quantified each day using MTT assay. The results were normalized to the mean optical density of the control for each day. MMS and cisplatin were obtained from Sigma-Aldrich®.

### Real-time quantitative PCR

Total RNA was extracted from cells using Quick-RNA kit (Zymo Research) according to the manufacturer’s instructions. Total RNA (1 µg) was subjected to reverse-transcription using qScript cDNA SuperMix (QuantaBio, VWR). Real-time quantitative PCR (RT-qPCR) were performed with the Roche LightCycler® 480 instrument and the PerfeCTa SYBR Green FastMix (QuantaBio, VWR) and were carried out in a final volume of 10 μL using 0.25 µL of each primer (25 μM), 5 μL of the supplied enzyme mix, 2.5 μL of H_2_O, and 2 μL of the template diluted at 1:10 [see Supplementary Table 1 for primer sequences]. After pre-incubation at 95 °C, runs corresponded to 35 cycles of 15 s each at 95 °C, 5 s at 60 °C, and 15 s at 72 °C. Melting curves of the PCR products were analyzed using LightCycler® software to exclude amplification of unspecific products. The results were normalized to different housekeeping gene transcripts (mouse RS9 or human 28S)^[30]^. 

### Immunoblotting

RIPA solution was used to extract whole-cell proteins. Cell extracts were analyzed after migration of 30 µg protein extract by Western blotting using a primary polyclonal antibody against Polκ (1/1000, Abcam ab57070). Protein quantifications were normalized with the β-actin signal (1/1000, Millipore).

### DNA microarray analysis 

Published DNA microarray datasets, GSE39582^[31]^ and GSE42284^[32]^, were reanalyzed for RIP140 and POLK expression using the Cancertool database^[33]^. A transcriptomic dataset from the TCGA-COAD RNA-seq data was also used^[4]^. Another published DNA microarray study obtained on a cohort encompassing 396 colon tumor samples with MSS and MSI CRC^[34]^ was also reanalyzed for RIP140 and POLK mRNA expression. Statistical significance was assessed using a Spearman correlation analysis. Correlation between *RIP140* and *POLK* gene expression was also studied using another cohort^[35]^. RNA sequencing data from the TCGA^[4]^ were reanalyzed using Cox proportional hazard regression^[36]^. RNAseq data obtained from CRC samples from the TCGA dataset were analyzed as described previously^[37]^. The Kaplan-Meier method was used to estimate overall survival (OS) calculated from the diagnosis until death. Patients lost to follow-up were censored at the time of last contact.

### Statistical analysis

All experiments were conducted independently at least three times. The results were expressed as the mean ± standard deviation (S.D.). Statistical comparisons were performed with Mann-Whitney or Spearman tests. For the Cancertool database analysis, a Pearson’s test was performed for the correlation analyses. A probability level (*P*-value) of 0.05 was chosen for statistical significance.

## RESULTS

### RIP140 regulates *POLK* gene expression in mouse models

**Figure 1:**
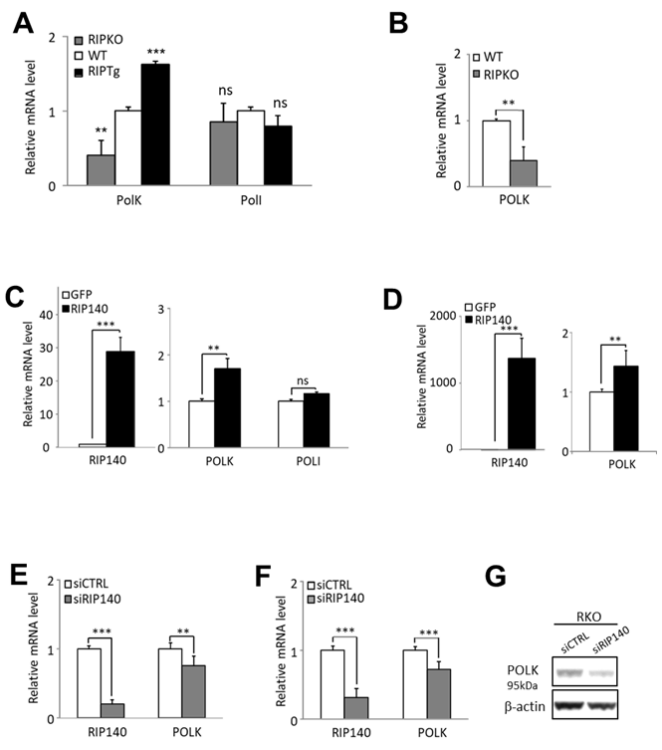
RIP140 regulates *POLK* gene expression. (A) RT-qPCR analysis of Polκ and PolI mRNA in the intestinal epithelium of mice lacking the Rip140 gene (RIPKO) and in transgenic mice overexpressing RIP140 (RIPTg) as compared to their WT littermates. The results for each gene are given in arbitrary units (AU) and expressed in fold change ± S.D. relative to WT after normalization to mouse RS9 mRNA. (B) The same as in (A) in immortalized MEFs from WT or RIPKO mice. (C) HCT116 cells were stably transfected with the pEGFP-RIP140-expressing vector (HCT-RIP140) or pEGFP alone (HCT-GFP). RIP140, POLK, and POLI mRNA levels were quantified by RT-qPCR. The results are expressed relative to GFP cells after normalization to human 28S mRNA. (D) The same as in (C) in HCT116 cells transiently transfected with a RIP140 expression vector. (E) RIP140 and POLK mRNA levels were quantified by RT-qPCR in HCT116 cells transiently transfected with siCTRL or siRIP140 siRNAs as indicated. The results are expressed as fold change ± S.D. relative to siCTRL after normalization to 28S mRNA (*n* = 3 independent experiments for each condition). (F) The same as in (E) performed in RKO CRC cells. (G) *POLK* expression analysis by Western blot of whole cell extract from RKO cells 48 h after transient siRNA transfection. Quantifications are expressed in AU after normalization to β-actin, used as a control of protein migration. A Mann-Whitney test was used for statistical analysis (ns = not significant, **P < 0.01, ***P < 0.001).

To explore the role of RIP140 on the regulation of the *POLK* polymerase gene expression, we first used transgenic mice in which the *Rip140* gene was either knocked out (RIPKO mice) or overexpressed (RIPTg mice) ^[24]^. As shown in Figure 1A, a significant decrease in the levels of *Pol*κ mRNA was observed in the intestinal epithelium of RIPKO mice, whereas an increase was noted in RIPTg mice as compared to wild-type animals (WT). These regulations appeared specific since the expression of other TLS polymerase genes from the Y subfamily such as *PolI* did not vary between the different genotypes [Figure 1A and data not shown]. The steady-state levels of Polκ mRNA were also significantly reduced in immortalized MEFs derived from the RIPKO mice as compared to cells isolated from WT animals [Figure 1B]. Altogether, these results demonstrate that RIP140 positively controls the expression of the Polκ gene in mouse cells and tissues.

### RIP140 regulates *POLK* gene expression in CRC cells

To check whether this regulation can be observed in human cells, we then analyzed the effect of RIP140 on *POLK* gene expression in human CRC cell lines. We confirmed the increased expression of the *POLK* gene at the mRNA levels in HCT116 cells either stably overexpressing RIP140 [Figure 1C] or transiently transfected with a RIP140 expression vector [Figure 1D]. We also unveiled that silencing the expression of the *RIP140* gene in HCT116, RKO or HCT29 CRC cells significantly affected *POLK* mRNA abundance [Figure 1E and F and Supplementary Figure 1] as well as the Polκ protein levels in RKO cells [Figure 1G].

### Transcriptional regulation of *POLK* gene transcription in CRC cells

**Figure 2:**
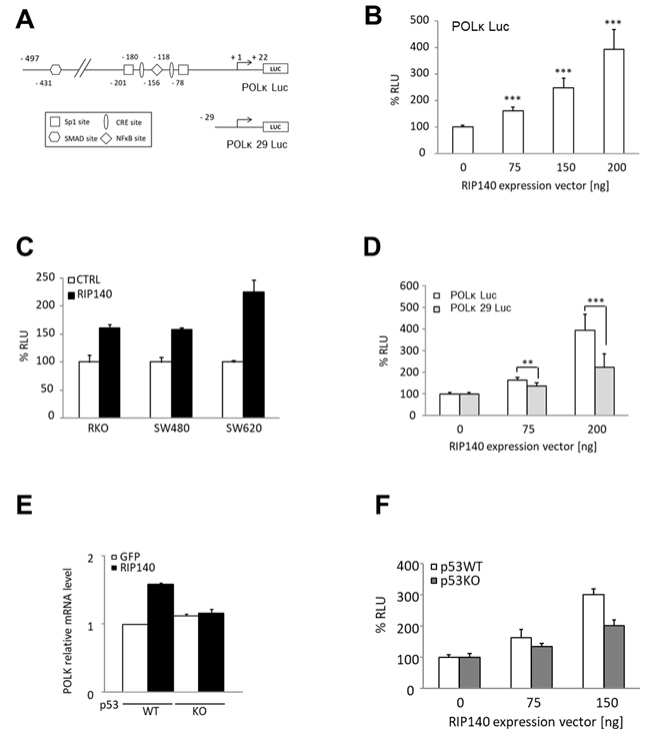
RIP140 regulates POLK at the transcriptional level in CRC cells. (A) Schematic representation of *POLK* proximal promoter cloned in a pGL3 promoter and the putative regulatory sites identified as well as the *POLK* 29 Luc deletion mutant. (B) The *POLK* Luc construct was transiently co-transfected into HCT116 cells with increasing doses of a pEF-cmyc-RIP140 expression vector and pRL-CMV-renilla as an internal control. The reporter activity is presented as relative luciferase activity (RLU) as mean ± S.D. (*n* = 3 independent experiments). (C) The same reporter assay as in (B) performed in RKO, SW480, and SW620 cells. (D) Luciferase reporter assay performed with the two reporter vectors described in (A), in HCT116 cells. Values, expressed as a percent of control, are means ± S.D. (*n* = 3 independent experiments). (E) The effect of RIP140 ectopic expression on POLK mRNA levels after transient transfection in p53WT or p53KO HCT116 cells. (F) Transactivation of the *POLK* gene promoter by increasing doses of a RIP140 expression vector in p53WT or p53KO HCT116 cells. A Mann-Whitney test was used for statistical analysis (**P < 0.01 and ***P < 0.001).

To decipher the mechanisms underlying the positive regulation of *POLK* gene expression by RIP140, we set up transient transfection experiments of HCT116 cells using the POLK Luc reporter construct encompassing the proximal promoter region of the *POLK* gene [Figure 2A]. As observed in Figure 2B, RIP140 significantly increased, in a dose-dependent manner, the luciferase activity driven by the *POLK* gene promoter, thus supporting a positive transcriptional regulation by RIP140. The same effects were observed in other CRC cells including RKO, SW480, and SW620 cells [Figure 2C].

### Mechanism of the transcriptional regulation by RIP140

Although RIP140 was first identified as a transcriptional repressor, we and others reported positive regulation of gene expression (for a review, see Ref.^[38]^). In particular, we described a transcriptional activation of gene expression by RIP140 through Sp1-mediated mechanisms^[39]^. Since the *POLK* gene promoter exhibits Sp1 binding sites, we tested if the deletion of these sites affected the transcriptional response to RIP140 ectopic expression. As shown in Figure 2D, the induction of luciferase activity by RIP140 was significantly reduced when we used a short reporter construct (*POLK* 29 Luc), suggesting that the regulation of *POLK* gene expression by RIP140 might be, at least in part, mediated by Sp1.

Interestingly, a close correlation between elevated POLK expression and p53 inactivation was previously reported in lung cancer tissues^[40]^. This effect is likely due to the direct role of p53 on the *POLK* gene since p53 strongly inhibits the *POLK* promoter activity in lung cancer cells, while this activity is much higher in p53^-/- ^MEF than in p53^+/-^ and p53^+/+^ MEFs^[40]^. Therefore, we checked whether the positive regulation of *POLK* gene expression by RIP140 was dependent on p53. By using HCT116 cells expressing or not the *TP53* gene, we found that, indeed, the regulation of the *POLK* gene by RIP140 was abolished in HCT116 p53^-/-^ cells when monitored at the mRNA level [Figure 2E] or using a luciferase reporter assay [Figure 2F], supporting that p53 is involved in the RIP140-mediated regulation of *POLK* levels.

### Correlation of gene expression in human CRC biopsies

**Figure 3:**
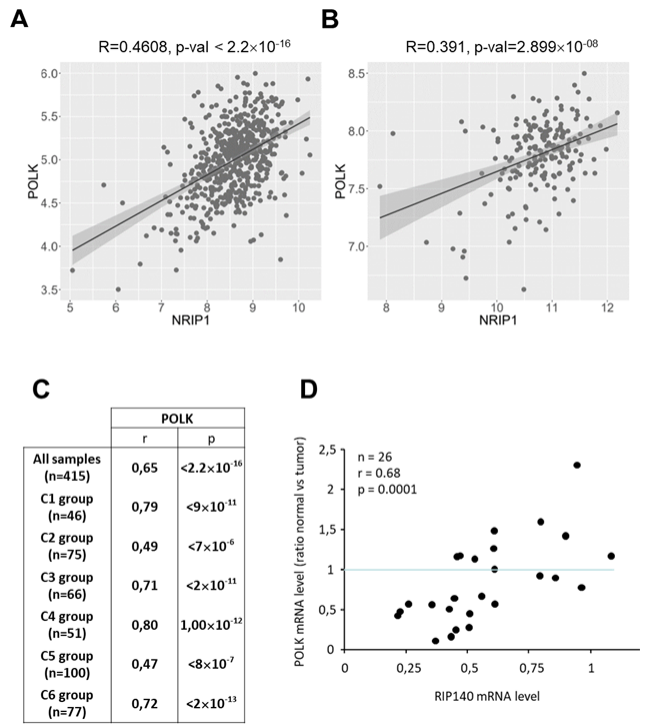
Correlation of *RIP140* and *POLK* gene expression in human CRC samples. (A) Analysis of *RIP140* and *POLK* correlation using the mRNAs expression from the Cancertool database in the GSE39582 cohort^[31]^. The correlation coefficient and the p-value (Pearson’s test) are indicated. (B) Same as in (A) with the cohort GSE42284^[32]^. (C) Correlations between *RIP140* and *POLK* gene expression performed with TCGA RNA-Seq cohort (*n* = 415)^[4]^ showing the correlations found in the different CRC molecular subtypes described in this cohort. (D) Correlation between *POLK* and *RIP140* gene expression in a cohort comparing the expression of “DNA replication” genes in CRC and in the adjacent normal mucosa (*n* = 26)^[35]^.

To validate in human CRC biopsies the expression data obtained in mouse tissues and in CRC cells *in vitro*, we used the Cancertool database^[33]^ to reanalyze the published Affymetrix DNA microarray data from two cohorts, namely GSE39582 containing 585 tumors^[31]^ and GSE42284 with 188 samples^[32]^. As shown in Figure 3A and B, and in perfect agreement with the data from mice, the results clearly show a significant positive correlation of *RIP140* mRNA levels with those of *POLK* in CRC biopsies. We also reanalyzed another transcriptomic dataset from the TCGA-COAD RNA-seq data^[4]^ obtained on 415 samples, which confirmed a significant correlation between RIP140 and POLK mRNA levels (r = 0.65; *P* < 2.2 × 10^-16^) [Figure 3C]. This dataset was further reanalyzed, taking into account the different molecular subtypes^[31]^. This allowed us to show a significant correlation of *RIP140* gene expression with that of *POLK* in the six different subgroups, in particular in the C2 group which corresponds to the MMR deficient subgroup. Finally, we confirmed these data in another cohort comparing the expression of “DNA replication” genes in CRC and in the adjacent normal mucosa^[35]^. We confirmed that *RIP140* gene expression strongly decreased in the tumor (data not shown) and that there was a strong correlation in the expression ratio (normal *vs. *tumoral) of the two genes (r = 0.68; *P* = 0.0001) [Figure 3D].

### Effect of the RIP^MSI^ mutation on the regulation of *POLK* gene expression

**Figure 4:**
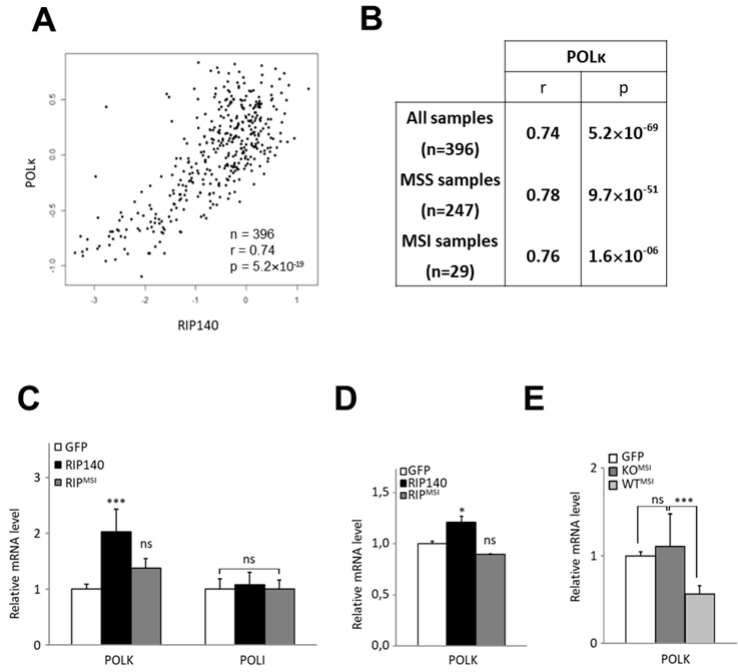
RIP140 and POLK in MSI CRC tumors. (A) Correlation between *RIP140* and *POLK* gene expression in 396 colorectal adenocarcinomas^[34]^. (B) Statistical significance was assessed using a Spearman correlation analysis on this cohort, containing 247 microsatellite stable (MSS) and 29 microsatellite instable (MSI) samples. Spearman correlation coefficients between *RIP140* and *POLK* gene expression are indicated for the whole cohort, as well as MSS and MSI samples. (C) mRNA quantification of the *POLK* and *POLI* genes in MEF RIPKO stable cells expressing either the GFP (white box) or the GFP fused wild-type form (black box) or the RIP^MSI^ mutant form (grey box) of RIP140. (D) mRNA quantification of the *POLK* gene in HT29 CRC cells transiently transfected with pEGFP, pEGFP-RIP140, or pEGFP-RIP^MSI^ expression vectors. (E) Analyses of the mRNA expression of the *POLK* gene in MEFs cells stably transfected with the human expression vector of RIP^MSI^ in a RIP140 wild-type background (MEF WT) or knock-out (MEF RIPKO) as compared to the control transfected with a GFP expressing vector in each condition. A Mann-Whitney test was used for statistical analysis (ns = not significant, *P < 0.05, **P < 0.01 and ***P < 0.001).

We recently demonstrated that RIP140 plays an important role in controlling microsatellite instability through the regulation of *MSH2* and *MSH6* genes^[26]^. To compare the correlation between *RIP140* and *POLK* gene expression in microsatellite stable (MSS) and instable (MSI) CRC tumors, we reanalyzed a transcriptomic dataset from 396 human CRC with both types of tumors^[34]^. As shown in Figure 4A, we observed a very significant positive correlation between *RIP140* and *POLK* mRNA levels in the whole cohort (r = 0.74; *P* = 5.2 × 10^-69^), thus confirming the results shown in Figure 3. Interestingly, *POLK* and *RIP140* gene expression were significantly correlated in both MSS and MSI tumors [Figure 4B]. 

In MSI CRC, we identified a frameshift mutation in the RIP140 coding sequence^[26]^. This mutation (called RIP^MSI^) generated a truncated protein which impaired the biological activity of the RIP140 protein. As shown in Figure 4C, the RIP^MSI^ protein was found to be less efficient than the WT protein to increase *POLK* gene expression. The same results were obtained on endogenous gene expression in HT29 CRC cells overexpressing wild-type or mutated RIP140 [Figure 4D]. Interestingly, the RIP^MSI^ mutant exhibited a dominant negative effect since its ectopic expression significantly decreased *POLK* mRNA accumulation only in WT MEFs, which express normal levels of RIP140, and not in RIPKO MEFs, which no longer express the *Rip140* gene [Figure 4E].

### Functional consequences of the regulation of POLK by RIP140

**Figure 5:**
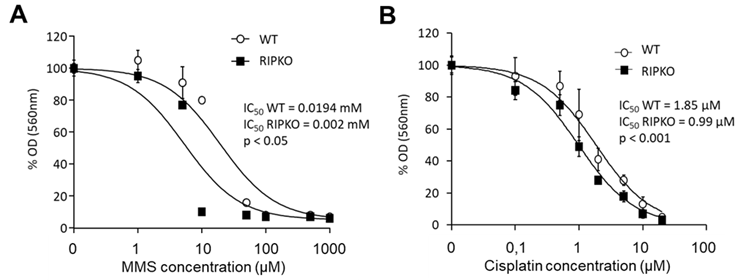
*RIP140* expression affects the response to cytotoxic drugs. (A) MEF WT and RIPKO cells were exposed or not to increasing doses of methyl methane sulfonate (from 0.5 µM to 1 mM). The optical density of diluted formazan crystals was expressed in percentage relative to the control. IC_50_ values of each cell type are mentioned together with the p-value of the nonlinear regression performed with GraphPad® software, allowing the comparison of IC50 between each cell type dose-response (IC_50_ ratio WT/RIPKO = 9.7, *P* < 0.05). (B) The same as in (A) with increasing doses of cisplatin (from 0.1 to 20 µM) (IC_50_ ratio WT/RIPKO = 1.9, *P* < 0.001). Mouse embryonic fibroblasts wild-type.

Since the *POLK* gene is involved in the cellular response to cytotoxic drugs including MMS and cisplatin through a TLS replication process^[41]^, we next measured the importance of RIP140 on MMS and cisplatin sensitivity by comparing IC_50_ ratios between RIPKO cells and their WT counterparts. As shown in Figure 5A and B, we observed a significant increase of sensitivity to MMS and cisplatin when the *RIP140* gene was knocked out (IC_50_ ratio WT/RIPKO = 9.7, *P* < 0.05 for MMS and IC_50_ ratio WT/RIPKO = 1.9, *P* < 0.001 for cisplatin). Altogether, these data suggest that the regulation of *POLK* gene expression by RIP140 can affect the cellular responses to various alkylating agents, including anticancer drugs.

### *POLK* expression impacts the prognosis value of RIP140 in CRC tumors

**Figure 6:**
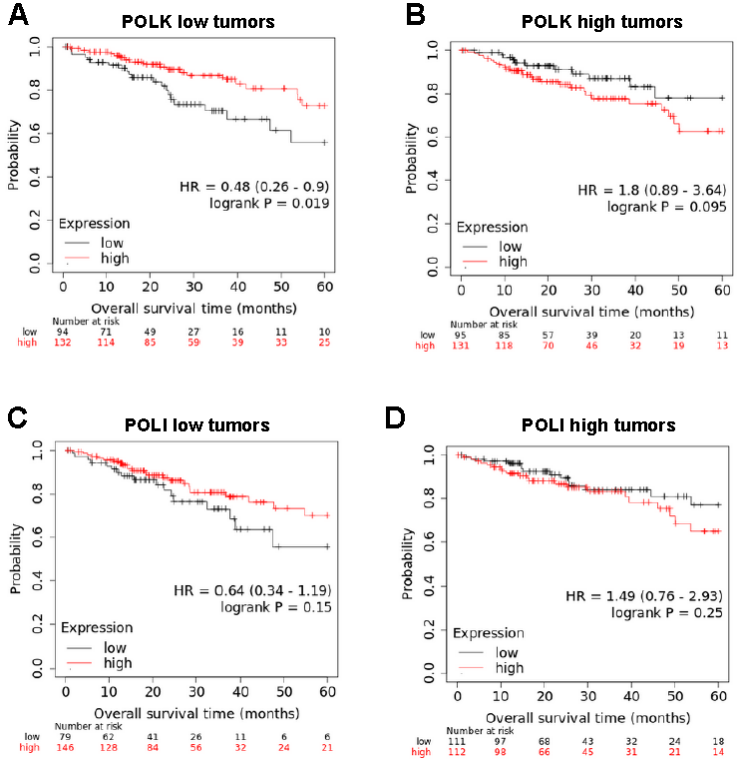
The prognosis value of RIP140 in CRC tumors is dependent on *POLK* gene expression. (A) Kaplan-Meier analysis of the cumulative OS of patients with low *POLK* gene expression was performed into the groups exhibiting low or high *RIP140* gene expression. A log-rank test was used for statistical analysis. (B) The same analysis as in (A) with tumors expressing high *POLK* gene expression. (C,D) A similar study was performed using *POLI* levels to define the two subcohorts.

Using the Kaplan-Meier Plotter database, we then investigated whether *POLK* levels contribute to the good prognosis value of RIP140 that we previously reported in CRC patients^[24]^. We reanalyzed RNAseq data obtained from 452 patients with colon adenocarcinoma that we separated into two groups of 226 patients, each with low and high *POLK* gene expression in the corresponding tumors using the median as a cutoff value. As shown in Figure 6A and B, a statistically significant association of high expression of RIP140 with a decreased risk of death in CRC patients was observed when their tumor exhibited low *POLK* gene expression [Figure 6A] but not in tumors with high *POLK *gene expression [Figure 6B]. As a control, no differences were observed when the 452-patient cohort was stratified based on the median value of *POLI* expression [Figure 6C and D]. Collectively, all the data obtained in this work strongly support a molecular and functional link between RIP140 and POLK in CRC.

## DISCUSSION

Colorectal cancer is a frequent neoplasm with high genomic instability including MSI due to defects in the MMR system. We previously described that the transcription factor RIP140 was a key factor in the regulation of intestinal homeostasis and tumorigenesis^[24]^. We also showed that RIP140 could be involved in microsatellite stability through the regulation of *MSH2/MSH6* gene expression^[26]^.

In the present study, we demonstrated that RIP140 also strongly regulates *POLK* gene expression, in both mouse and human cells and tissues. Data obtained using luciferase reporter assays demonstrate that this regulation took place at the transcriptional level and implicated the proximal region of the *POLK* gene. Several transcription factors including p53^[40]^, Sp1, and CREB^[29]^ or AhR^[42]^ have been shown to control the expression of the *POLK* gene, and some of them have their activity regulated by RIP140^[43]^. The data presented herein suggest that p53 is a good candidate to mediate, at least partly, the regulation of *POLK* gene expression since the positive effect of RIP140 is lost in HCT116 cells no longer expressing the *TP53* gene. Moreover, it has been shown that the HDAC inhibitor Trichostatin A was able to induce *POLK* gene expression^[29]^. Since RIP140 strongly interacts with HDACs^[44]^, it is possible that HDAC sequestration out of the *POLK* gene promoter also participates in the positive effect exerted by RIP140 on *POLK* gene transcription as already demonstrated for other transcription factors^[39]^. Further work will be necessary to fully decipher the mechanisms used by RIP140 to transcriptionally control *POLK* gene expression.

Cell survival relies on a subtle equilibrium between accurate genomic DNA replication and less stringent DNA synthesis often linked to DNA damage. POLK plays an important and critical role in such equilibrium by contributing to several important DNA transaction pathways. These include translesion synthesis, which allows stalled replication forks to bypass a lesion and restart^[45]^; the replication checkpoint^[46]^, a crucial pathway that regulates the replication stress response to a large array of insults that cause replication arrest; and the synthesis of intrinsic non-B microsatellite DNA^[7]^. Consistent with these multiple and complex roles, both overexpression of *POLK* (associated with advanced disease stages and shorter survival in patients with glioma^[47]^ and non-small cell lung carcinomas^[48]^) and under-expression of *POLK* (frequently observed in colorectal, lung, stomach, and breast cancers^[29,35,49,50]^) have been documented to lead to genetic instability. Indeed, excessive Polκ can interfere with fork progression and trigger a mutator phenotype and chromosome instability, while downregulation of Polκ expression can affect fork progression and induce replicative stress. Since both situations can confer a selective growth advantage during cancer cell evolution, this underlines the importance of tight regulation of *POLK *gene expression at the transcriptional level for the maintenance of genome integrity. Therefore, our discovery here establishing the role of RIP140 in *POLK* gene regulation not only may explain why POLK is abnormally expressed in cancer, notably in CRC, but can also clarify why cancers cells respond differentially to anticancer genotoxic drugs that target DNA replication forks. This last point is illustrated in the present work by cell sensitivity to MMS treatment, which can be modulated by RIP140. 

In addition, it is worth mentioning that the regulation of *POLK* gene expression could intensify the phenotypic consequences of the regulation of *MutSα* gene expression by RIP140^[26]^. Indeed, this TLS polymerase displays a high accuracy during dinucleotide microsatellite DNA synthesis and has been proposed to play a role in the maintenance of microsatellite stability^[7]^. Moreover, Polκ interacts with MSH2^[12]^ and could therefore participate in the control of MutSα activity. Defining the precise involvement of *POLK* gene induction in the effect of RIP140 on the maintenance of genome integrity in colon cancer cells (in particular on the stability of microsatellites) and on their resistance to anticancer drugs will obviously require further work. It would also be interesting to decipher whether, as it is suggested for the MSH2 protein^[51]^, POLK could be involved in the RIP140-mediated transcriptional regulation of gene expression. 

Altogether, the present data suggest that *POLK*-defective cells exhibit an altered DNA replication program, thus explaining the spontaneous genetic alterations observed in *POLK*-deficient mice^[8]^. Transcriptional deregulation of *POLK* gene expression may therefore participate in intestinal tumorigenesis and account, at least in part, for the tumor suppressor role of RIP140 that we previously suggested.

## DECLARATIONS

### Copyright

© The Author(s) 2022.
