# Supplementary material for: RIP140 regulates POLK gene expression and the response to alkylating drugs in colon cancer cells
Source: Cancer Drug Resist. 2022 May 7;5(2):401–14. doi: 10.20517/cdr.2021.133 (PMC9255241; doi:10.20517/cdr.2021.133)
Supplement: Supplementary file 1 [file cdr-5-2-401-SupplementaryMaterials.pdf]

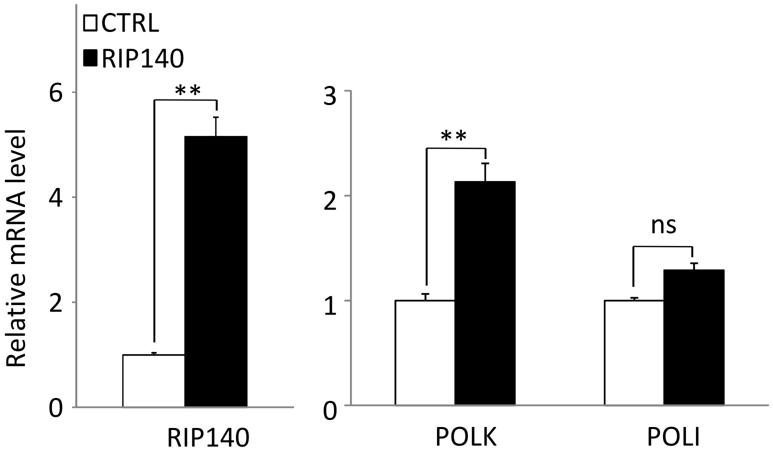

Supplementary Figure 1. Expression of POLK in human HT29 CRC cells. mRNA quantification of RIP140, POLK and POLI genes by RT-qPCR in HT29 cells after transient transfection of pEF-cmyc-RIP140.

Supplementary Table 1. Primer sequences

Gene Symbol	Forward Sequence	Reverse Sequence
mRIP140	AGAACGCACATCAGGTGGCA	GATGGCCAGACACCCCTTTG
mPOLK	AGCCTGGCTTCCGATTCT	TCCATGATGCAAACAGGGTA
mPOLI	AGGGCCTATGGACTGCTACC	TGTGAGAGTCTTCCGTGTGG
hRIP140	AATGTGCACTTGAGCCATGATG	TCGGACACTGGTAAGGCAGG
hPOLK	CCTCCTGGGAGTTGTAGTCG	CCCATCCTAAGCAGAAGATCA
hPOLI	GGTCGTGAGAGTCGTCAGTG	ATCAACCATTGGGGTCATCA
